# Characteristics and Distribution of Organic Phosphorus Fractions in the Surface Sediments of the Inflow Rivers around Hongze Lake, China

**DOI:** 10.3390/ijerph17020648

**Published:** 2020-01-19

**Authors:** Jie Wan, Xuyin Yuan, Lei Han, Hongmeng Ye, Xiaofan Yang

**Affiliations:** 1 Key Laboratory for Integrated Regulation and Resources Development on Shallow Lakes, Ministry of Education, College of the Environment, Hohai University, Nanjing 210098, China; wanjie@ahpu.edu.cn (J.W.); hanlei8613@163.com (L.H.); 2 College of Biological and Chemical Engineering, Anhui Polytechnic University, Wuhu 241000, China; xiaofan108@ahpu.edu.cn; 3 Fujian Key Laboratory of Eco-Industrial Green Technology, Wuyishan University, Wuyishan 354300, China; hongmengye@sina.com

**Keywords:** sediment, organic phosphorus, inflow river, Hongze Lake, eutrophication

## Abstract

In this study, the characteristics and distribution of the organic phosphorus (Po) fractions in the surface sediments of seven inflow rivers around Hongze Lake in China were analyzed with a soil Po fraction method, as used by Ivanoff. The relationships between the Po fractions and physiochemical features of sediments were also discussed. The results showed that, the sediments of the rivers had been moderately pollution with certain ecological risk effects except the Waste Yellow River. The relative contribution order of the Po fractions in the sediments was residual Po > HCl-Po > fulvic acid-Po > humic acid-Po > labile organic phosphorus (LOP). Moderately labile organic phosphorus (MLOP) was the main part of the Po forms in the whole sediments. The risk of phosphorus released from river sediments was the highest in the western region, followed by the southwestern region, and finally the northwestern region. There were significant correlations between Po forms and total phosphorus (TP), inorganic phosphorus (Pi), and Po. Non labile organic phosphorus (NLOP) had the strongest correlation with TP. The distribution of Po forms in each region was different due to the impact of human activities, industrial and agricultural production and the land types; the heaver polluted sediments with higher Po fractions. It is suggested that most of the sediments of the inflow rivers in the regions have certain ecological risk effects and P of them have an important contributions on the eutrophication of Hongze Lake. Po forms can provide a reliable theoretical basis for dealing with the change of water quality and should be paid more attention in the lake eutrophication investigation. There was reciprocal transformation between different Po forms, especially non-bioavailable fraction can change into bio-available ones. The results can provide a basis for the earth cycle of phosphorus and a new perspective of eutrophication control of shallow lakes.

## 1. Introduction

Phosphorus (P) is an essential nutrient for aquatic organisms and is a limiting nutrient for primary production in lake ecosystems [1,2,3], so excessive phosphorus (P) is a key factor for eutrophication [4]. Over the last few decades, the external inputs of P have been gradually reduced; however, the release of internal P from the sediments of lakes to the overlying water has become a significant source of P [5,6]. These may cause continuous eutrophication in lakes, even after control of external inputs [7]. Therefore, the various chemical interconversions of phosphorus, biological effectiveness, the exchange between the sediments and overlying water have been widely studied [8,9]. However, not all the fractions of phosphorus can be released from sediments, and cause lake eutrophication [10,11,12]. Accordingly there are many extraction methods for phosphorus fractions in sediments [13]. Most of them divide the fractions into two parts: inorganic phosphorus (Pi) and organic phosphorus (Po) [14]. The Pi fraction is composed of exchangeable phosphorus, Fe, Al, Mn oxides-bound phosphorus and Ca-bound phosphorus. Po refers to various Po compounds which include nucleic acids, phospholipids, inositol phosphates, sugar phosphates, condensed P, etc. [15]. However, most former studies have mainly focused on the compounds of Pi. In fact, the internal source of Po is the important part for constituents of P [16]. Microbial degradation and mineralization of Po compounds play a vital role in the migration and transformation of P, and may result in Po becoming Pi in the overlying water to participate in the geochemical cycle [17]. A large number of studies show that the relative contribution of Po in the sediments can reach 80.0% of total P [18]. However, until now, investigations of the species, quantification, concentrations, dynamics, biogeochemical cycling and ecological significance of these organic P compounds have been limited due to its complexity and the limitations of analytical methods [19].

For these reasons, in recent years, sequential extraction schemes have been developed based on the assumption that chemical extractants selectively dissolve discrete groups of Po [20]. These methods were first used in soils then in sediments. Bowman and Cole [21,22] developed the first integrated scheme for separating soil Po into four distinct fractions: labile Po, moderately labile Po, moderately resistant Po, and highly resistant Po. Hedley et al. [23,24] used a simple method that rapidly separated and analyzed Pi and Po from environmental samples into several fractions, and then quantized the microbial biomass-P. Then Ivanoff et al. [25] divided Po into three fractions: labile Po; moderately labile Po, containing HCl-Po and fulvic acid-P; and non-labile Po containing residual Po and humic acid-P. The method greatly increased the Po recovery by using additional steps and prolonging the extraction time for each Po fraction. The pH value of fulvic acid-Po and humic acid-Po was reduced to 0.2, which made the separation more strict and had no effect on the recovery of organic phosphorus. This also increased the analysis of residual Po. Fan et al. modified some aspects of the Bowman Cole system. They changed the division of highly resistant Po and moderately resistant Po according to pH 1.0–1.8. This method can significantly increase the content of labile Po and resistant Po, but decrease the content of moderate Po, which may be related to the order of extraction. Up to now there has been no standard fractionation system for Po in sediments [26].

In the last twenty years, the booming economy of China, with fast development of agricultural and industrial activities, has led to serious eutrophication of the hydroecological sytem [27,28]. The second national investigation surveyed 138 lakes with an area of more than 10 km^2^, and the results showed that 85.4% of the lakes have eutrophication issues, and almost half of them (>40%) are hyper-eutrophic lakes [29]. Wu et al. investigated 22 typical lakes of China, and found that 59.1% of the lakes presented a certain degree of eutrophication [30]. Hongze Lake is the fourth largest freshwater lake in China [31]. Experts at home and abroad have done a lot of work in the aspects of pollution source control, total amount of nutrients in sediment, distribution of the nutrient elements forms, sediment transport and dredging etc., but the lake eutrophication is still serious. In recent years, agriculture and aquaculture around Hongze Lake have developed rapidly; traditional agriculture and aquaculture promote the accumulation of phosphorus in sediments. As a lake, Hongze Lake has become the intersection of the main and tributaries of the upper reaches of the river basin [32]. In view of this, it is not comprehensive to only pay attention to the nutrient elements in the water body and sediments of the lake itself. The characteristics and distribution of nutrient elements especially the limiting nutrient P fractions in the sediments of the rivers flowing into the lake should be studied. The characteristics and distributions P forms in the sediments of the rivers flowing into the lake directly affects characteristics and distribution P fractions the in the sediments of the lake, thus affecting the eutrophication level of the lake. Based on our investigation, there are few researches on the fractions of P in the sediments of the rivers flowing into the lake, let alone the systematic research and analysis on the relationship between Po forms in the sediments of the rivers flowing into the lake through the land use types [33,34,35]. Therefore, it is of great significance to study the characteristics and distribution of Po components in the sediment of the inflow rivers around Hongze to basis for eutrophication control in lakes and geochemical cycle of phosphorus.

The objectives of the present study were to: (1) investigate physiochemical features in the surface sediments of inflow rivers around Lake Hongze; (2) investigate the characteristics and distribution of Po fractions and the bioavailability in the surface sediments of the study areas according to the land use type sediments, and (3) analyze the relationships between Po and physicochemical nature of the sediments.

## 2. Materials and Methods

### 2.1. Study Area

**Figure 1:**
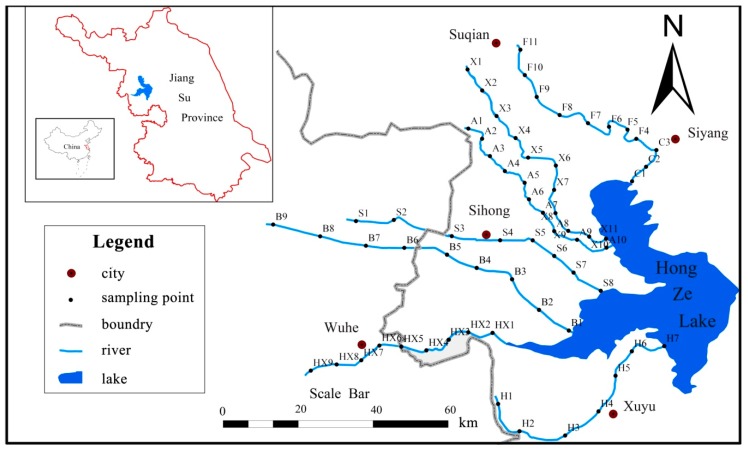
The map of sampling points. North western rivers: Waste Yellow River (F and C); Xuhong River (X); and an River (A); western rivers: Sui River (S); and Bian River (B); south western rivers: Huaihongxin River (HX); and Huaihe River (H).

We take the northwest to southwest of the Hongze Lake Basin as the research area. Seven tributaries or main stream sediments were selected in the basin as the research objects. The distribution of sampling points is shown in Figure 1.

Hongze Lake, located in the northwest of Jiangsu Province (33°06′–33°40′ N, 118°10′–118°52′ E), is a shallow water lake with a water area of 1597 km^2^, a maximum water depth of 4.37m, an average water depth of 1.77 m, a water storage capacity of 27.9 × 108 m^3^ and a shoreline of 354 km. The lake area has monsoon climate characteristics, which is regulated by the Hongze Lake water body, with four distinct seasons. The annual average precipitation is 925.5 mm, and the rainy season is mainly from June to September [36,37]. There are six counties along the lake, namely Xuyi, Hongze, Sihong, Siyang, Huaiyin, and Jinhu. The rivers entering the lake, which include the Huaihe River, Sui River, Huaihongxin River, Xinbian River, and Xuhong River, are mainly concentrated in the west. The rivers leaving the lake mainly include Huaishuxin River, Subei irrigation channel, Huaihe River water channel, and Huaihe River water channel, of which the water volume entering the river accounts for 60–70% of the total water volume leaving the lake. The annual average flow into the lake is 33 billion m^3^, and the water exchange rate reaches 11 times per year. As a hub connecting the middle and lower reaches of the Huaihe River, Hongze Lake and Huaishuxin River connect the Huaihe River system with the Yi, Shu, and Sishui systems. It is necessary to not only regulate and control the flood in the upper and middle reaches, but also to store part of the incoming water, which irrigates a large area of farmland in Northern Jiangsu, and protects the safety of more than 20 million people and 2 million square hectares farmland in the Lixia River area in Northern Jiangsu. The east line of the South to North Water Transfer Project in China will also transfer 400 m^3^/s of water northward through Hongze Lake. However, with the aggregation of human activities, Hongze Lake has become the main receiving water body of industrial wastewater and domestic sewage in the upper and middle reaches of the Huaihe River, and water pollution has reduced the ecological service function of Hongze Lake [38,39].

### 2.2. Sediment Sampling and Analysis

In this study, 65 sampling points were set up using a grasp sampler in June 2015 and 2016, with a sampling depth of 0–10 cm. the sampling points numbered C1 toF11 are located on the Waste Yellow River, X1to X11 are on the Xuhong River, A1 to A10 are on the An River, S1 to S8 are on the Sui River, B1 to B9 are on the Bian River, XH1 toXH9 are on the Huihongxin River, and H1 to H7 are on the Huaihe River. Each sediment sample (a composite of samples from five nearby sampling sites) was immediately put into air-sealed plastic bags and storage in a heat preservation box with ice (about 4 °C). Then, the collected sediments were freeze-dried, crushed, passed through standard 100-mesh sieves and stored at 4 °C in the dark until further analysis. Sediment pH was determined on sediments suspended in deionized water at a ratio of 1:2.5 [40]. Total Fe, Al, Mg, Mn, and Ca were detected by a PW2440 type ray fluorescence analyzer from Panaco (Panaco, Almelo, Netherlands). The organic matter (OM) was measured after treatment with K_2_Cr_2_O_7_/H_2_SO_4_ according to Walkey–Black method [41]. Total nitrogen (TN) used the concentrated H_2_SO_4_ digestion method [42]. After addition of 5 mL 1M H_2_SO_4_ and 0.5 g of K_2_S_2_O_8_ and autoclave digestion during 30 min at 121 °C, total phosphorus (TP) in the extract was determined [43]. Inorganic phosphorus (Pi) was checked by direct extraction with 1 M HCl (16 h), and then analyzed through colorimetry using the molybdate blue method and organic P (Po) in the extract was calculated as the difference between total P and inorganic P [44].

### 2.3. Sequential Fractionation of Sediment Po

**Figure 2:**
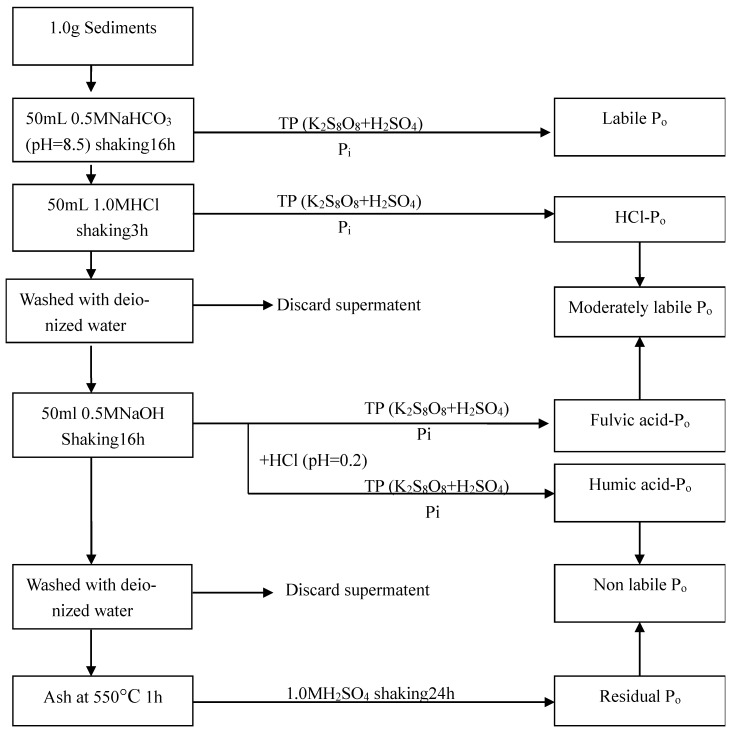
Fractionation procedure of organic phosphorus (Po) fractions in the sediments.

A sequential extraction method used to obtain different forms of Po based on the Ivanoff et al. scheme [45], presented in Figure 2. Po is divided into labile Po, moderately labile Po, and non-labile Po fractions. Firstly, labile Po was extracted with 0.5M NaHCO_3_ at pH 0.5. The extracted Po was mainly called the loosely adsorbed Po on the sediment colloids. The moderately labile Po was extracted with 1.0M HCl followed by 0.5 M NaOH. The NaOH extract was acidified to pH 0.2 with 37% HCl, and the non-labile component (humic acid-Po) was isolated from the appropriate fraction (fumic acid-Po). Finally, by buffering the residue extracted by NaOH, the non-labile segmentation is determined by high resistance H_2_SO_4_ dissolved to 1 M at 550 °C for 1 h. Each extraction step is performed at room temperature in an orbital shaker running at 4500 r/min. TP in all extracts was calculated with an aliquot that was digested with K_2_S_2_O_8_ and H_2_SO_4_. Po in the samples was calculated by the difference between TP and Pi.

### 2.4. Statistical Analyses

The whole samples were analyzed in triplicate and the results are expressed as the average values. The distribution of sampling points was drawn by ArcGIS (ESRI, Redlands, CA, USA). The descriptive statistical analyses were conducted using the SPSS ver. 23.0 statistical software package (IBM, Armonk, NY, USA). Charts were plotted using the Origin 9.0 software (OriginLab, Northampton, MA, USA).

## 3. Result and Discussion

### 3.1. Sediment Characteristics

The general characteristics and physiochemical features of lake sediments are shown in Table 1.

The average value of the four metal elements in the study area was Al > Ca > Fe > Mn. The average value of Ca ranged from 3.38% to 6.03%, with the order Bian River > Sui River > Huaihongxin River > Huaihe River > An river > Xuhong River > Waste Yellow River. The concentrations of Ca in Hongze Lake basin were higher than other basins in the middle of China, such as the Tiaoxi basin [46,47]. This may be related to the large amount of calcium carbonate deposition in the soil of the study area [48]. The spatial variation of trace elements in the sediments of Mn was the largest, followed by Fe. The spatial variations of trace elements in the sediments were related to the different soil patterns, hydrodynamic forces, geographical environments and the different land use patterns, such as land for farming, also influenced the concentrations of them [49,50]. As a whole, the sediments of the seven rivers were weakly alkaline. The mean values of pH varied from 7.47 to 8.59. The order is the same as Ca. pH was significantly correlated with Ca (R = 0.914, *p* < 0.01, n = 65). Carbonate calcium is greatly affected by environmental pH, which easily forms precipitation in alkaline environments and is easy to dissolve and release in acid environments.

OM is one of the most important colloids in sediments, mainly derived from many kinds of residual of authigenic alga and bacteria. It can absorb, distribute, and complex with heavy metals and organic pollutants, which is an important indicator to reflect the organic nutrition level of sediment [51,52]. The order of OM is Bian River > Sui River > An river > Huaihong River > Huaihe River > Waste Yellow River > Xuhong River, with the range from 0.89% to 2.11%.TN ranged from 840.96 mg·kg^−1^ to 1130.62 mg·kg^−1^; TP ranged from 488.90 mg·kg^−1^ to 960.22 mg·kg^−1^. OM, TN, and TP showed a great spatial difference. However, the sequence of OM, TN, and TP was the same.TN and TP also had a positive correlation with OM (R = 0.827, R = 0.697, *p* < 0.01, n = 65). Concentrations of OM, TN, and TP in the sediments were consistent with the trophic status of those studied lakes. In sediments of the seven rivers, the pollution was more and more serious from northwest to west and from southwest to west. Bian River was the most polluted, then the Sui River. This was because the rivers of the west were far away from the cities. Along the rivers, aquaculture and agriculture are relatively developed. Domestic sewage and industrial waste water are not centrally disposed.

Exogenous input is the main factor affecting TP and TN, and the concentrations were higher in the heavily polluted areas. According to the Sediment Quality Guidelines issued by the Ministry of Environment and Energy of Ontario (Canada), when the concentration levels of TP exceeds 600 mg·kg^−1^ and TN exceeds 600 mg·kg^−1^ in the sediment, it can cause the lowest level of ecotoxicity effect [53]. With the conclusion of Liu [54], when TP < 500 mg·kg^−1^ in the sediment, there had been no pollution; when 500 mg kg^−1^ < TP < 1000 mg·kg^−1^ in the sediment, there had been moderate pollution, so in addition to the Waste Yellow River, the sediments of rivers from the northwest to the southwest of Hongze Lake Basin had a certain ecological risk effect and had been moderate pollution with certain ecological risk effects. For the whole region, the nitrogen/ phosphorus (N/P) of the sediments ranged from 1.17 to 1.72, far less than the Redfield ratio (C: N: P = 106:16:1). On the one hand, this reflects that the phosphorus in the sediments is mainly imported from land. Due to the different geological structure, landform, land use type, vegetation damage, and surface runoff erosion, a large number of phosphorus-containing substances are brought in by land-based sources of rivers entering the lake. On the other hand, this reflects that the biochemical action of the sediment the sediment–water interface in shallow lakes is intense, and the dissolved oxygen with low concentration causes nitrogen (N) to be lost due to mineralization and degradation, while P is enriched in the sediments [55,56].

### 3.2. The Characteristics and Distribution of Phosphorus Fraction in Sediments

#### 3.2.1. TP, Pi, Po Fractionations in Sediments

**Figure 3:**
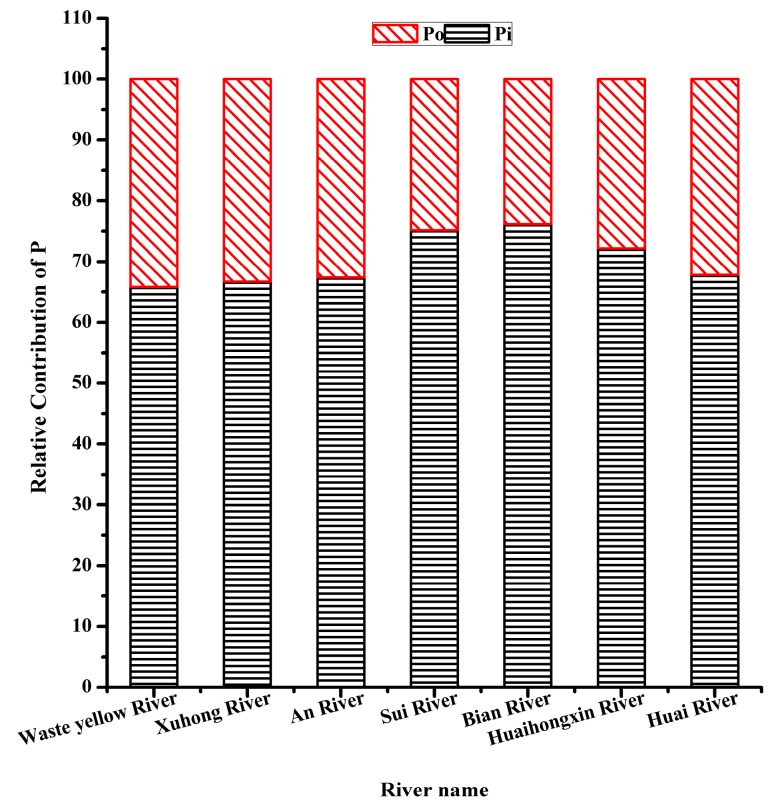
Concentrations of P fractions in the sediments of the seven rivers. Organic phosphorus (Po), inorganic phosphorus; (Pi), phosphorus (P).

**Figure 4:**
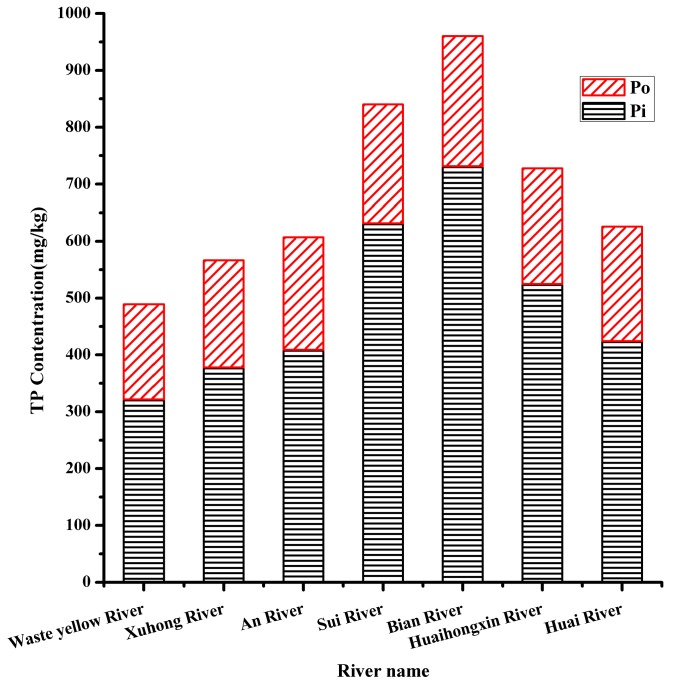
Relative contribution of P fractions in the sediments of the seven rivers. Total phosphorus (TP).

From Figure 3 and Figure 4, Pi was the main content of TP, ranging from 321.74 mg·kg^−1^ to 731.34 mg·kg^−1^, accounting for 65.81–76.15% of TP. Po ranged from 167.16mg·kg^-1^ to 228.88 mg·kg^−1^. Relative contributions were 34.19%, 33.30%, and 32.61% for Waste Yellow River, Xuhong River, and An River, respectively. The population mean was 33.84% for the northwestern region. It was the highest among the southwestern region, named Huaihongxin River (27.85%) and Huaihe River (32.17%) with the population mean at 30.18% and the western region, including Sui River (24.82%) and Bian River (23.83%) with the lowest population mean at 24.58%. 

Except for point B1, the concentration of TP at the entrance of each river to the lake was the highest value of the river. One reason was that the dynamic condition of the lake inlet was poor, and the sediments were easy to accumulate, which lead to the increase of the concentration of TP. The higher concentrations of TP in the sediments of the lake inlet will affect the concentrations of TP in the water of Hongze Lake, which will finally increase the risk of eutrophication of Hongze Lake. The concentration of TP in the sediment of B1 was lower, it may because that there was a large amount of land used for growing grass. Grassland can reduce the concentration of nutrients in sediment [47]. The increase of TP concentration in each river was accompanied by the increase of Pi and Po concentration, but the percentage of Po in TP decreased with the increase of TP concentration. This was consistent with the results of Jin’s study [57], and the proportion of Po in relatively clean sediments is relatively high. This fully explained why a part of Po had transformed into Pi, and on the other hand, there were some external source inputs in the rivers. The different distributions of TP, Pi, and Po were related to pollution sources and land patterns. The western region was the most seriously polluted of all, as along these two rivers there were mainly rural areas or the suburbs of towns where agriculture was the main activity and there was no sewage collection system for domestic sewage and scattered industrial wastewater; they discharged into the river in a non-point-source way. The land use type was mainly cultivated land and grassland, long-term unreasonable fertilization and improper disposal of livestock manure, resulting in serious agricultural non-point source pollution. In addition, due to a large number of cage cultures in the rivers, with the increase of organic food, animal and plant residues and other nutrients and organic matters, the river sludge increased, the nutrient load of sediment was heavy, and most of the water body was eutrophicated. The northwestern region was polluted the least. Although the three rivers were near the city Suqian or town Siyang, some of the sample points were inside the city or town, such as F10, F11, etc. The density of the urban population and the development of industry might bring a mounting of exogenous P input, but the point-source pollutions in the city and town were collected and controlled better for disposal. Along the Waste Yellow River and Xuhong River there were some protective wetlands and primary protection areas of water sources. In addition, the upper reaches of Waste Yellow River collected the river called Chenzi River; it had abroad riparian zone and wide river surface. A large hydrodynamic force, strong self-purification capacity and light nutrient load of sediment in the river area might be the cause of the observed lower concentration of P [58]. Not long before our sampling, sediment dredging had been carried out on the Xuhong River. This might another reason for the lower of concentration of P. The southwestern region was the second. The water quality of Huaihe River has been improved through years of treatment. However, the Huaihongxin River is near the western region, and the industrial structure, lifestyle, and sewage discharge around it were similar to those in the western region.

#### 3.2.2. Po Fractionation in Sediments

Po is mainly deposited in the processes of river input, human emission, metabolism of aquatic animals, and plants. Its content will directly affect the availability level of soluble P required by primary productivity [59]. Po can be used to roughly estimate the formation and degradation of organic matter. It is a better indicator of eutrophication than TP [60]. The organic phosphorus in sediment can be divided into labile organic phosphorus (LOP), moderately labile organic phosphorus (MLOP), and non-labile organic phosphorus (NLOP) composed residual Po and fumic acid–Po. Distributions of organic phosphorus in the sediments of seven rivers are shown in Table 2: There recovery of Po ranged from to 95.45% to 106.57%, with an average of 101.6%. This indicated Po in sediments was sufficiently extracted with this method.

LOP is the most active component of Po, which has high biological efficiency. However, this part of phosphorus was also the least in the form of Po. Its percentage content was the key factors for the degradation of Po mineralization to Pi [61]. The LOP of sediments ranged from 10.47 mg·kg^−1^ to13.55 mg·kg^−1^, and the average was 11.81 mg·kg^−1^, the only relative contribution was 5.66% to6.52%, and the average only 5.94% of Po, less than 7% in the soil [62]. The sequence of percentage content of LOP in river sediments was Waste Yellow River > Xu River > An River > Huaihe River > Huaihongxin River > Sui River > Bian River. The region order was northwestern > southwestern > western.

Obviously, the higher the pollution degree, the lower the contributions of LOP. The difference of contribution of LOP indicated that the contribution of polluted sediment released to overlying water through mineralization is higher than that of less polluted sediment, which has a greater impact on eutrophication of rivers [63].

MLOP included HCl–Po and fulvic acid-Po. It is the most active component in the Po, which has a certain biological effectiveness. On the whole, the concentration of MLOP ranged from 87.51 mg·kg^−1^ to 105.20 mg·kg^−1^, with the relative contribution of Po at 43.93% to 54.47%. MLOP was the same as LOP, where the concentration was higher in heavily polluted sediments, and the percentage decreased. HCl–Po was the main part of the MLOP, the chemical components are mainly phosphate ester, phospholipid, nucleic acid, phosphoprotein, and phosphosaccharide, which are easily, decomposed bio-macromolecules with poor stability. Under certain conditions, they can be hydrolyzed or mineralized, decomposed into soluble small molecule organic phosphorus or soluble phosphate, and have potential biological effectiveness through migration and diffusion of pore water [64,65]. In the study area, a higher polluted degree with higher concentration, ranged from 36.07 mg·kg^−1^ to 79.78 mg·kg^−1^, accounting for 22.45–33.32% of Po. Huang Qinghui et al. found that the release of acid extractable organic phosphorus from sediments may be one of the important processes leading to lake eutrophication in the study of Lake Taihu, Chaohu, and Longgan in the east of China. Hua Liping et al. also put forward the same view in the study of Lake Baiyangdian in the north [66]. In addition, some studies have shown HCl–Po/Po can reflect the difference of lake eutrophication levels [67]. HCl–Po/Po for Sui River and Bian River both exceeded 0.3, the highest of the other regions. This can further indicates that HCl–Po may be an important source of phosphorus for rivers, which can well reflect the eutrophication level of rivers, and provide a new theoretical basis for the establishment of aquatic environment quality standards and the assessment and prediction of lake eutrophication in the future [68]. Fulvic acid-P has relatively weak potential bioavailability due to its solubility. The release of it under anaerobic conditions is a process of Po mineralization under the mechanism of microbial enzymology. It accounted for 10.62–32.02% of Po.

NLOP was the least active component of Po, with low bioavailability. It consisted of humic acid–Po and residual Po with relative contributions of 7.71–13.41% and 25.61–42.70% of Po. The concentration and the percentage of NLOP increased with the increase of pollution degree, it was different from LOP and MLOP. The NLOP relative contribution of Bian River was over 50% to Po.

In this study, the relative contribution of bio-available organic phosphorus (LOP + MLOP) [69] in rivers with light pollution is higher than that in rivers with heavy pollution, and the increase of the bio-available phosphorus concentration in water becomes potential endogenous phosphorus pollution in lakes, which is consistent with other research results. In general, the risk of phosphorus released from river sediments was the highest in the western region, followed by the southwestern region and finally the northwestern region. The MLOP is the main form of Po of the sediments in the northwestern and southwestern region. However in the western region the NLOP was the capital form of Po. This may be because the western region had the highest OM. In the high organic matter, the organic phosphorus components that can be degraded and deposited by organic matter are far-reaching and mineralized. In the case of weak water exchange capacity, they are absorbed by particulate matter and enter the sedimentary facies in the form of organic matter, resulting in changes in the composition of the sediment, so most of the refractory organic phosphorus components are easy to deposit [70]. Zhang et al. [71] found that the inactive organic phosphorus is only a relative chemical solubility, the main components such as phytate phosphorus can be absorbed and utilized by microorganisms and plants, and still have potential biological activity under certain conditions. The concentration of NLOP in the seriously polluted river is higher than that in the lightly polluted river, which shows that the non-active organic phosphorus also has an important impact on lake eutrophication. The order of the relative contribution order of organic phosphorus in each river was residual Po > HCl-Po > fulvic acid–Po > humic acid–Po > LOP with the ratio of 5.77:5.14:3.04:1.86:1.

#### 3.2.3. Relationships between Po Fractions and Physiochemical Features

The relationships between Po fractions and physiochemical features are shown in Table 3:

There was a significant correlation between Ca and MLOP (R = 0.911, *p* < 0.01, n = 65). It might be that MLOP mostly existed in the form of calcium. NLOP had a significant correlation with Fe (R = 0.933, *p* < 0.01, n = 65). This showed that NLOP was easily an effective carrier of Fe [72]. There were significant correlations between Po fractions and TP, Pi, and Po. This showed that the source of phosphorus in sediment was consistent. Among them, the correlation between NLOP and TP was higher (R = 0.934, *p* < 0.01, n = 65), than with the Pi (R = 0.930, *p* < 0.01, n = 65). This proved the viewpoint of Zhang [71]: NLOP was an important component of total phosphorus, which can be converted into Pi under certain conditions. It was a potential phosphorus source of sediment. We can further analyze whether NLOP is the carrier of Fe from Corum, K.W.’s atomistic modeling of mineral–water interfaces theory [73], which is one of the important reasons for NLOP to become a potential phosphorus source. The main mechanism is a problem to be further studied.

Po fractions were also strongly positively correlated with OM and TN. This was consistent with previous results [74]. Generally speaking, the organic phosphorus in the lake ecosystem mainly comes from the mixed input of the terrestrial, marine, and authigenic Po, and the Po in the sediment is mainly controlled by the OM. The study on Wuli Lake in Taihu Lake [74] showed that in the early diagenesis process of the sediment, the organic phosphorus was released preferentially when the internal OM in the lake degraded, and the degradation was as follows: The OM can be released or even released to the organic matter during the early diagenesis. It is similar to the application of organic fertilizer in the soil, which can increase the soil Po and redistribute the soil OM pool. The cation exchange capacity and the Po with lower activity form may be released. The high correlation of mass fraction is due to the fact that the organic exchange group in the surface colloid of sediment is mainly composed of humic acid, while LOP provides more organic exchange groups and forms more exchangeable organic–inorganic complexes on the surface of sediment. Humic acid binding P does not belong to bio-available phosphorus, which is not necessarily related to the occurrence of lake eutrophication.

## 4. Conclusions

(1) The sediments of the whole rivers had been moderately pollution with certain ecological risk effects, except the Waste Yellow River. Except for point B1, the concentration of TP at the entrance of each river to the lake was the highest value of the river. It is suggested the higher concentrations of TP in the sediments of the lake inlet will finally affect the concentration of TP of Hongze Lake, which will lead to a risk of eutrophication of Hongze Lake. (2) The characteristics and distribution of Po fractions were different in rivers with different pollution levels, suggesting that Po fractions were indicators of eutrophication and should be paid more attention in an- lake eutrophication investigation. (3) Non labile organic phosphorus (NLOP) had the strongest correlation with TP. It may also suggest that NLOP can transfer into a potential source of available P for aquatic phytoplankton and bacteria, although it was considered as the non-bioavailable fraction.

In the process of eutrophication treatments of shallow lakes that rivers flow into, more solicitude should be shown for non-point source pollution and internal phosphorus of the lake. However, the contractions of Po fractions and the transformation of different forms of Po in the sediments of the upstream rivers can’t be neglected, which should be paid more attention. What’s more the transformations between the Po fractions are complex. We can use 31P-NMR to research the mechanism of the transformation of internal Po in the sediments in the further investigation to help controlling the eutrophication. Additionally, the concentrations of phosphorus forms in the sediment of the inflow rivers have a certain contribution to the eutrophication of the shallow water lake, but the specific contribution degree needs to be further fitted with the water quality model.

## Figures and Tables

**Table 1:** Characteristics and physiochemical features of sediments. The organic matter (OM), total nitrogen (TN), total phosphorus (TP), inorganicphosphorus (Pi), organic phosphorus (Po), nitrogen/phosphorus (N/P). Coefficient of Variation (C/V).

Region	River Name	Parameters	CaO (%)	MnO (%)	Fe_2_O_3_ (%)	Al_2_O_3_ (%)	pH	OM (%)	TP (mg·kg^−1^)	TN (mg·kg^−21^)	N/P
Northwestern	Waste Yellow River	Range	3.2–3.56	0.04–0.10	1.58–4.35	4.12–8.66	6.49–8.17	0.11–2.87	413.65–597.94	686.92–930.41	1.52–1.91
		Mean	3.38	0.07	3.15	6.39	7.47	1.22	488.90	840.96	1.72
		CV (%)	3.42	28.10	24.64	20.54	5.00	61.69	12.00	10.00	0.07
	Xuhong River	Range	3.14–4.63	0.04–0.10	1.91–4.68	6.64–11.18	7.03–8.2	0.19–2.99	450.57–692.42	554.32–1020.42	1.20–1.99
		Mean	4.10	0.07	3.48	8.91	7.65	0.98	566.40	887.60	1.57
		CV (%)	11.72	27.12	22.30	14.73	4.40	78.78	15.00	17.00	0.13
	An River	Range	3.31–4.81	0.04–0.10	2.29–5.06	8.1–12.65	7.47–8.02	0.47–3.00	372.71–934.86	699.29–1220.22	1.30–1.88
		Mean	4.26	0.07	3.68	9.24	7.67	1.39	606.75	967.83	1.60
		CV (%)	8.65	22.59	14.96	12.96	2.30	46.29	20.00	14.00	0.1
	Population mean		3.91	0.07	3.44	8.18	7.60	1.20	554.02	898.80	1.63
Western	Sui River	Range	4.79–6.28	0.06–0.11	4.2–5.35	11.08–15.63	7.92–8.71	0.84–3.46	649.15–1051.45	1051.85–1243.00	1.12–1.62
		Mean	5.89	0.08	4.45	13.80	8.34	1.73	840.22	1130.62	1.35
		CV (%)	7.70	21.42	11.98	8.89	3.34	45.11	18.00	5.00	0.13
	Bian River	Range	4.79–6.47	0.08–0.13	4.19–6.22	11.27–15.81	7.83–9.04	0.99–3.92	799.76–1076.54	813.42–1367.89	0.95–1.33
		Mean	6.03	0.10	5.34	14.02	8.59	2.11	960.22	1123.82	1.17
		CV (%)	7.42	19.03	9.52	9.54	4.10	35.32	10.00	16.00	0.11
	Population mean		5.96	0.09	4.90	13.91	8.47	1.92	900.22	1127.22	1.26
Southwestern	Huaihongxin River	Range	4.05–5.55	0.05–0.11	2.72–5.40	8.71–13.25	7.78–8.35	0.77–3.04	630.21–793.88	673.65–1316.54	0.84–2.09
		Mean	5.06	0.08	4.02	11.22	8.07	1.68	727.51	1039.27	1.43
		CV (%)	9.40	22.59	17.40	10.53	2.90	43.13	8.00	20.00	0.27
	Huaihe River	Range	3.21–4.71	0.05–0.10	2.29–5.06	8.1–12.65	7.20–8.11	0.89–2.32	547.99–689.67	960.54–1120.31	1.51–1.67
		Mean	4.33	0.07	3.77	10.74	7.80	1.54	625.48	1015.81	1.62
		CV (%)	11.10	25.00	22.79	11.93	3.51	28.04	8.00	6.00	0.05
	Population mean		4.695	0.075	3.895	10.98	7.935	1.61	676.5	1027.54	1.525

**Table 2:** Distributions and the recovery of different Po fractions in the sediments. Labile organic phosphorus (LOP) moderately labile organic phosphorus (MLOP) and non labile organic phosphorus (NLOP), total extracted phosphorus (OPEX).

Region	Sediments	LOP		MLOP				NLOP				OPEX (mg·kg^−1^)	Po (mg·kg^−1^)	Recovery (%)
		NaHCO_3_-P_O_		HCl-Po		Fuvic Acid-Po		Residual Po		Humic Acid-Po				
		(mg·kg^−1^)	%	(mg·kg^−1^)	%	(mg·kg^−1^)	%	(mg·kg^−21^)	%	(mg·kg^−1^)	%			
Northwestern	Waste Yellow River	10.47	6.52	36.07	22.45	51.44	32.02	41.14	25.61	21.54	13.41	160.67	167.16	96.12
	Xuhong River	11.22	6.12	49.90	27.22	45.00	24.55	54.47	29.71	22.74	12.40	183.34	188.59	97.22
	An River	11.29	5.98	56.26	29.78	40.52	21.45	57.34	30.36	23.48	12.43	188.89	197.90	106.57
	mean	10.99	6.20	47.41	26.49	45.66	26.01	50.98	28.56	22.59	12.75	177.63	184.55	99.97
Western	Sui River	12.53	5.66	73.43	33.19	29.31	13.25	84.96	38.40	21.03	9.50	221.26	208.56	106.09
	Bian River	13.55	5.66	79.78	33.32	25.42	10.62	102.24	42.70	18.46	7.71	239.45	228.88	104.62
	mean	13.04	5.66	76.61	33.25	27.36	11.93	93.60	40.55	19.75	8.61	230.36	218.72	105.35
Southwestern	Huaihongxin River	12.01	5.75	69.13	33.09	30.02	14.37	72.52	34.72	25.20	12.06	208.89	202.63	103.09
	Huai River	11.58	5.90	62.63	31.92	35.80	18.25	63.05	32.14	23.13	11.79	196.19	201.19	97.52
	mean	11.80	5.83	65.88	32.51	32.91	16.31	67.79	33.43	24.17	11.93	202.54	201.91	100.30
Population mean		11.81	5.94	61.25	30.51	36.34	18.11	68.81	34.28	22.21	11.06	200.74	199.80	101.60

**Table 3:** The relationships between Po fractions and physiochemical features.

Fractions	MnO	Fe_2_O_3_	Al_2_O_3_	CaO	pH	OM	TN	TP	Po	Pi
Liable Po	0.759 **	0.713 **	0.658 **	0.671 **	0.743 **	0.600 **	0.578 **	0.756 **	0.723 **	0.696 **
Moderately labile Po	0.682 **	0.627 **	0.564 **	0.911 **	0.875 **	0.595 **	0.572 **	0.673 **	0.633 **	0.658 **
Nonlabile Po	0.759 **	0.933 **	0.864 **	0.575 **	0.662 **	0.689 **	0.680 **	0.934 **	0.930 **	0.511 **

** *p* < 0.01; n = 65.
